# Evolution of Photoluminescence, Raman, and Structure of CH_3_NH_3_PbI_3_ Perovskite Microwires Under Humidity Exposure

**DOI:** 10.1186/s11671-018-2470-0

**Published:** 2018-03-07

**Authors:** Rubén Segovia, Geyang Qu, Miao Peng, Xiudong Sun, Hongyan Shi, Bo Gao

**Affiliations:** 1 Institute of Modern Optics, Key Lab of Micro-optics and Photonic Technology of Heilongjiang Province, Key Laboratory of Micro-Nano Optoelectronic Information System, Ministry of Industry and Information Technology, Department of Physics, Harbin Institute of Technology, Harbin, 150001 China; 2 School of Chemistry and Chemical Engineering, Harbin Institute of Technology, Harbin, 150001 China; 3 Collaborative Innovation Center of Extreme Optics, Shanxi University, Taiyuan, 03006 China

**Keywords:** CH_3_NH_3_PbI_3_ perovskite microwires, Photoluminescence, Raman, X-ray diffraction, Humidity

## Abstract

Self-assembled organic-inorganic CH_3_NH_3_PbI_3_ perovskite microwires (MWs) upon humidity exposure along several weeks were investigated by photoluminescence (PL) spectroscopy, Raman spectroscopy, and X-ray diffraction (XRD). We show that, in addition to the common perovskite decomposition into PbI_2_ and the formation of a hydrated phase, humidity induced a gradual PL redshift at the initial weeks that is stabilized for longer exposure (~ 21 nm over the degradation process) and an intensity enhancement. Original perovskite Raman band and XRD reflections slightly shifted upon humidity, indicating defects formation and structure distortion of the MWs crystal lattice. By correlating the PL, Raman, and XRD results, it is believed that the redshift of the MWs PL emission was originated from the structural disorder caused by the incorporation of H_2_O molecules in the crystal lattice and radiative recombination through moisture-induced subgap trap states. Our study provides insights into the optical and structural response of organic-inorganic perovskite materials upon humidity exposure.

**Electronic supplementary material:**

The online version of this article (10.1186/s11671-018-2470-0) contains supplementary material, which is available to authorized users.

## Background

Hybrid halide perovskite CH_3_NH_3_PbX_3_ (X = I^−^, Br^−^, and Cl^−^) semiconductors have emerged with great impetus in the past years owing to their easy and low-cost fabrication through low-temperature solution processes without any sophisticated or vacuum equipment needed. In addition, their distinguished optical and electronic properties make these materials suitable for optoelectronic applications [1–3]. Methylammonium lead iodide (CH_3_NH_3_PbI_3_, MAPbI_3_) has been the most studied material in the hybrid halide perovskite family, with the majority of the previous investigations focused on thin films for application in photovoltaic cells as light harvesting [4–7]. Besides thin films for solar cells, low-dimensional isolated MAPbI_3_ crystals with regular morphologies, such as microwires (MWs) [8], nanowires [9], microrods [10], microdisks [11], and nanoplatelets [12], also synthesized through solution processing but with different crystallization routes, are promising for micro/nanoscale optoelectronic and photonic devices. In particular, wire structure has some advantages in comparison with thin films like large surface-to-volume ratio, fewer grain boundaries, and lower defect/trap density [13] and lasing action [14], along with better charge separation and conductivity [15]. In recent years, the application of MAPbI_3_ micro- and nanowires in optoelectronic devices has increased notably due to the implementation of different preparation methods [8–10]. For example, because of the high sensitivity to visible light, high-photoluminescence (PL) quantum efficiency, long photocarrier diffusion length, and optical gain, perovskite wires have been used in the fabrication of photodetectors [8, 13, 16, 17], lasers [14, 18], and optical waveguides [19]. Moreover, one-dimensional nanowires applied in solar cells showed faster carrier separation and higher lateral conductivity than the bulk MAPbI_3_ form [15].

Nevertheless, material stability, highly related to life durability and device performance, is one of the major problems in organic-inorganic perovskite semiconductors. Degradation due to humid ambient air is a key issue. In the presence of water vapor, MAPbI_3_ forms an intermediate monohydrate phase and/or dihydrate phase, then decomposes into the precursor materials lead iodide (PbI_2_) solid and aqueous methylammonium iodide (CH_3_NH_3_I, MAI), and ultimately, MAI could further decompose into volatile methylamine (CH_3_NH_2_), hydrogen iodide (HI), and iodide (I_2_) [20–26].

Although the degradation process in hybrid perovskites is well known and with the recent increment in the use of perovskite MWs in photonic devices, as far as we know, there are no studies about the effect of humid ambient air in the optical properties and structure of MAPbI_3_ MWs. The response of this material under a humid environment can affect the performance of perovskite microwire-based optoelectronic devices. Therefore, herein, we have investigated MAPbI_3_ MWs upon exposure to humidity in the dark using PL, Raman spectroscopy, and X-ray diffraction (XRD). The evolution of the spontaneous emission, vibrational, and structural properties of MAPbI_3_ MWs was observed for several weeks. Our study shows that, in addition to the common hybrid perovskite degradation, humidity induced enhancement and redshift in the MWs photoemission, and slight variations in the Raman bands and XRD peak positions. We relate these changes to trap-assisted radiative recombination through defects within the bandgap induced by moisture and to crystal structure modifications due to the infiltration of H_2_O molecules into the material.

## Experimental

### Synthesis of CH_3_NH_3_PbI_3_ Microwires

MAI was synthesized by adding dropwise 40 ml of hydroiodic acid (HI) (55–58 wt% in water, Aladdin) into 30 ml of methylamine (CH_3_NH_2_, 30–33 wt% in methanol, Aladdin) in a round flat-bottom flask in ice bath, along with magnetic stirring for subsequent 2 h. Then, the solution was heated at 90 °C on a hot plate for 3 h for the evaporation of the solvents, obtaining a pale brown powder. Next, the pale brown powder was washed and filtered three times with ethanol and dried in an oven at 60 °C overnight, obtaining white MAI powder. MAPbI_3_ MWs were prepared by a one-step solution self-assembly method [11].

#### Sample Preparation for PL and Raman Measurements

MAPbI_3_ precursor solution was synthesized by mixing 50.7 mg of MAI and 50.9 mg of PbI_2_ (99.9%, Aladdin) in 5 ml of *N*,*N*-dimethylformamide (DMF) (99.9%, J&K Scientific Ltd.) at 60 °C for 20 min and sonicating for 10 min, obtaining a yellowish solution. Then, for the crystallization of the microwires, 20 μl of the precursor solution was deposited on a 2.5 × 2.5 cm^2^ glass slide, which was placed on a stage in a beaker. The beaker was filled with dichloromethane (DCM, CH_2_Cl_2_, 99.5%; Fuyu Fine Chemical) below the stage and was covered with film (Parafilm M), and then it was placed in an oven at 65 °C for 3 h.

#### Sample Preparation for XRD Measurements

Sample preparation for XRD measurements was made with the same procedure described above, with the difference that 24.7 mg of MAI and 72.3 mg of PbI_2_ were mixed in 3 ml of DMF and 50 μl of this solution was used for the microwires crystallization step.

### Exposure to Humid Air

The as-prepared MAPbI_3_ MW samples were placed in an airtight container with a calibrated hygrometer and stored inside a cabinet in the dark, with a room temperature ~ 20 °C. In the first 4 weeks, the humidity was given by natural weather conditions, being 45 ± 5% relative humidity (RH) in the first 3 weeks and 55 ± 5% RH for the fourth week. From the fifth week, humid air was induced with a salt-saturated solution. For this, a small open holder with natural salt and deionized water was placed in the airtight container beside the samples, providing a stable atmosphere of 80 ± 2% RH. The samples were only taken out from the cabinet for PL, Raman, and XRD characterization when required.

### Photoluminescence and Raman Spectroscopy

PL and Raman measurements of MWs were performed with a Renishaw InVia spectrometer. PL spectra were obtained with a 633 nm excitation light and ~ 5 μW laser power. Raman spectra were taken with an excitation wavelength of 532 nm and a laser power of 16 μW. For both techniques, the acquisition time was 10 s, and a × 50 objective lens (numerical aperture (NA) = 0.75) was used to focus and collect the light in a backscattering configuration. All the spectra were collected in ambient conditions (~ 20 °C, ~ 30% RH).

### X-ray Diffraction

XRD patterns were obtained with a PANalytical X’Pert Pro Multipurpose diffractometer equipped with a Cu-Kα (*λ* = 1.5418 Å) radiation source, operated at 40 kV and 40 mA, using a step size of 0.026° and a time per step of 0.2 s over an angle range of 5°–70°. XRD was performed in ambient conditions (~ 20 °C, ~ 30% RH).

### Scanning Electron Microscope and Optical Microscope Characterization

SEM image was acquired with a Hitachi SU8010 cold field emission electron microscope and the optical image with the Olympus BX51 microscope through a × 20 objective (NA = 0.40).

## Results and Discussion

### MAPbI_3_ Microwires

**Fig. 1:**
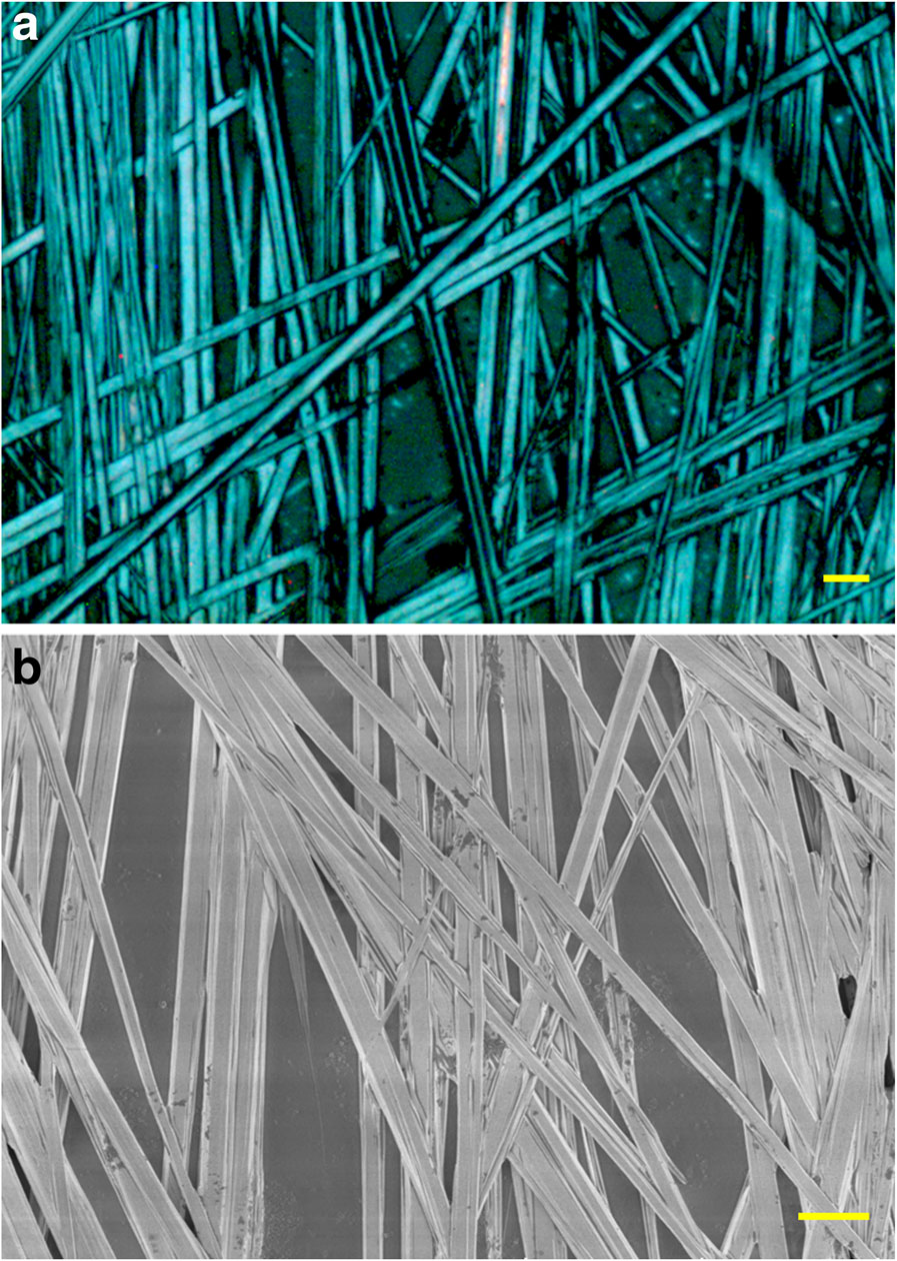
**a** Optical microscope and **b** SEM image of MAPbI_3_ microwires on glass slide. Scale bars represent 10 μm

MAPbI_3_ MWs were prepared by a one-step solution self-assembly method [11], in which an antisolvent (DCM) vapor diffuses into the MAPbI_3_ solution (MAI and PbI_2_ in DMF solvent), assisting in the crystallization and growth of the MWs. The morphology of the as-prepared MAPbI_3_ MWs was characterized by an optical microscope and SEM. As shown in Fig. 1, the crystallization produced long, straight, and mostly interlaced MWs, with a length ranging from a few millimeters to centimeters and a width of 2–5 μm. Furthermore, the MWs were dispersed on almost the whole glass slide substrate. The XRD pattern of the as-prepared MAPbI_3_ MWs and its comparison with the precursor materials and a reference patterns is shown in Additional file 1: Figure S1. As shown in Additional file 1: Figure S1, the strong diffraction peaks observed at 2*θ* values of 14.11°, 28.45°, 31.90°, and 40.48° can be assigned to (110), (220), (310), and (224) crystal planes of the tetragonal perovskite structure [2, 27]. The calculated lattice parameters *a* = *b* = 8.8703 Å and *c* = 12.6646 Å also indicate a tetragonal crystal structure of the MAPbI_3_ MWs (see Additional file 1: Table S1 for calculated data), which is in good agreement with previous studies [1, 2]. In such perovskite structure, the MA^+^ is located in the center of the crystal and a [PbI_6_]^−^ octahedron in each corner of the tetragonal structure [2].

### Photoluminescence Evolution of MAPbI_3_ Microwires Under Humidity

To evaluate the impact of humidity on the MAPbI_3_ MWs spontaneous emission, PL spectroscopy was performed along 11 weeks. Because of the inhomogeneity in the MWs (see Additional file 1: Figure S2), we measured ten different MWs (nine MWs the last week) chosen randomly each week, thus obtaining a general view about the response of the spontaneous emission at different stages of moisture exposure. In the first 4 weeks, the RH in the sample storage place was the same as the local weather conditions, being 45 ± 5% in the first 3 weeks and rising to 55 ± 5% in the fourth week. Then, from the fifth to the 11th week, the RH of 80 ± 2% was controlled with a salt-saturated solution (as described in the “Experimental” section). PL measurements were carried out with a red laser (*λ* = 633 nm) and at low excitation power (~ 5 μW) in order to avoid the local heating and damage of the MWs by high laser intensities. The degradation by high laser intensities has been observed in polycrystalline MAPbI_3_ films [28, 29], which is mainly due to the low thermal conductivity of MAPbI_3_ [30]. Moreover, short acquisition time (10 s) was used to reduce the sample exposure to the laser light to avoid thermal decomposition and to minimize light soaking (defects curing effect) by trap filling from photogenerated free-charge carriers and O_2_, which could reduce the nonradiative recombination channels and increases the PL intensity [10]. This material healing phenomenon could hide the surface and bulk defects that humidity may cause in the material.

**Fig. 2:**
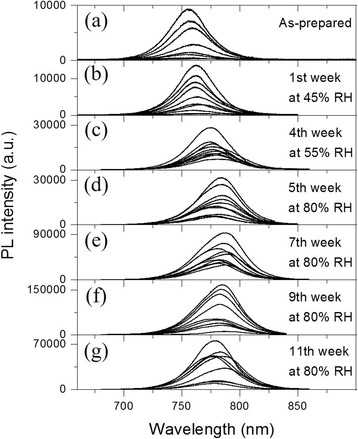
Photoluminescence spectra of MAPbI_3_ MWs at different stages of humidity exposure. **a** As-prepared sample, after the **b** 1st week at 45% RH, **c** 4th week at 55% RH, and **d** 5th, **e** 7th, **f** 9th, and **g** 11th weeks at 80% RH. All spectra taken in ambient conditions, with an excitation wavelength of 633 nm, a laser power ~ 5 μW, an acquisition time of 10 s, and a laser spot diameter of ~ 1 μm on the sample

PL emission evolution of the perovskite MWs is shown in Fig. 2. All the PL spectra present a single emission peak along the different stages of humidity exposure. For the as-prepared MWs (Fig. 2a), the PL peaks are centered around 759 nm, which is in good agreement with MAPbI_3_ polycrystalline thin films [31, 32], microwires [8], nanowires [9], and other irregular morphologies [9] fabricated by solution processes. After the first week at 45% RH (Fig. 2b), the PL peaks shifted to ~ 763 nm, and then in the fourth week at 55% RH (Fig. 2c), the peaks shifted to ~ 777 nm. From the fifth week in which the MWs were at 80% RH (Fig. 2d–g), the PL peaks stabilized to a value of ~ 780 nm. These results show that the spontaneous emission of the MWs shifted towards longer wavelengths upon humidity exposure, with the overall PL peaks redshifted by ~ 21 nm. The PL peaks at ~ 759 nm of the as-prepared MWs correspond to an optical energy bandgap (*E*_g_) value of 1.63 eV, whereas after the 11 weeks of moisture exposure, the peaks at ~ 780 nm correspond to an *E*_g_ value of 1.59 eV. The possible degraded product PbI_2_, the monohydrate phase, and the dihydrate phase present an *E*_g_ value of 2.5, 3.10, and 3.87 eV, respectively [21, 33, 34]. Therefore, the shift of the emission peaks after moisture exposure was not due to these byproducts but should be attributed to the MAPbI_3_ MWs.

As shown in Fig. 2, although the MWs present both high and low PL intensities in each stage of humidity, the overall intensity increased from the fourth week to the ninth week and decreased in the 11th week but still higher than the first weeks. This indicates that the radiative and nonradiative recombination rate changed and that the exposure to humidity resulted in the reduction of the nonradiative recombination channels. In previous studies on MAPbI_3_ thin films, the PL enhancement has been reported using post-fabricated treatments, such as sample exposure to direct water vapor streams for seconds [35] or to 35% RH for 4 h and 65% RH for 30 min [36], which was attributed to the passivation of bulk and surface defects by H_2_O molecules. However, the redshift of the PL peak was not observed, probably because the films were exposed to lower RH and less time than our MW sample or because the effect of humidity in thin films and MWs is different. Besides that, chemical and structural defects can acts as trap-assisted recombination centers for the photoexcited charge carriers [35, 37]. These trap states (i.e., vacancies, interstitials) are energy levels within the bandgap and can be deep and shallow traps [38]. Deep trap states, energy levels away from the band edges, are responsible for nonradiative recombination pathways [38]. Shallow trap states, energy levels close to the valence band (VB) and conduction band (CB), can act as radiative recombination channels and emit photons with less energy than those related with the CB-to-VB transition, leading to a redshift of the PL emission [39, 40]. In addition, it has been proposed that only shallow traps are formed in the surface of MAPbI_3_ thin films when reacting with H_2_O molecules [22]. For these reasons, we suggest that in our experiments, the trap-assisted nonradiative recombination centers (deep-level defects) were passivated by humidity, and thus the overall MWs PL intensity was enhanced. Nevertheless, humidity did not passivate the trap-assisted radiative recombination centers (shallow-level defects), but increased them, and consequently the MWs PL redshifted upon moisture exposure. These defects in the crystal structure created by humidity could alter the atomic positions and so modify the vibrational properties of the MWs, which can be observed by Raman spectroscopy.

### Raman Evolution of MAPbI_3_ Microwires Under Humidity

**Fig. 3:**
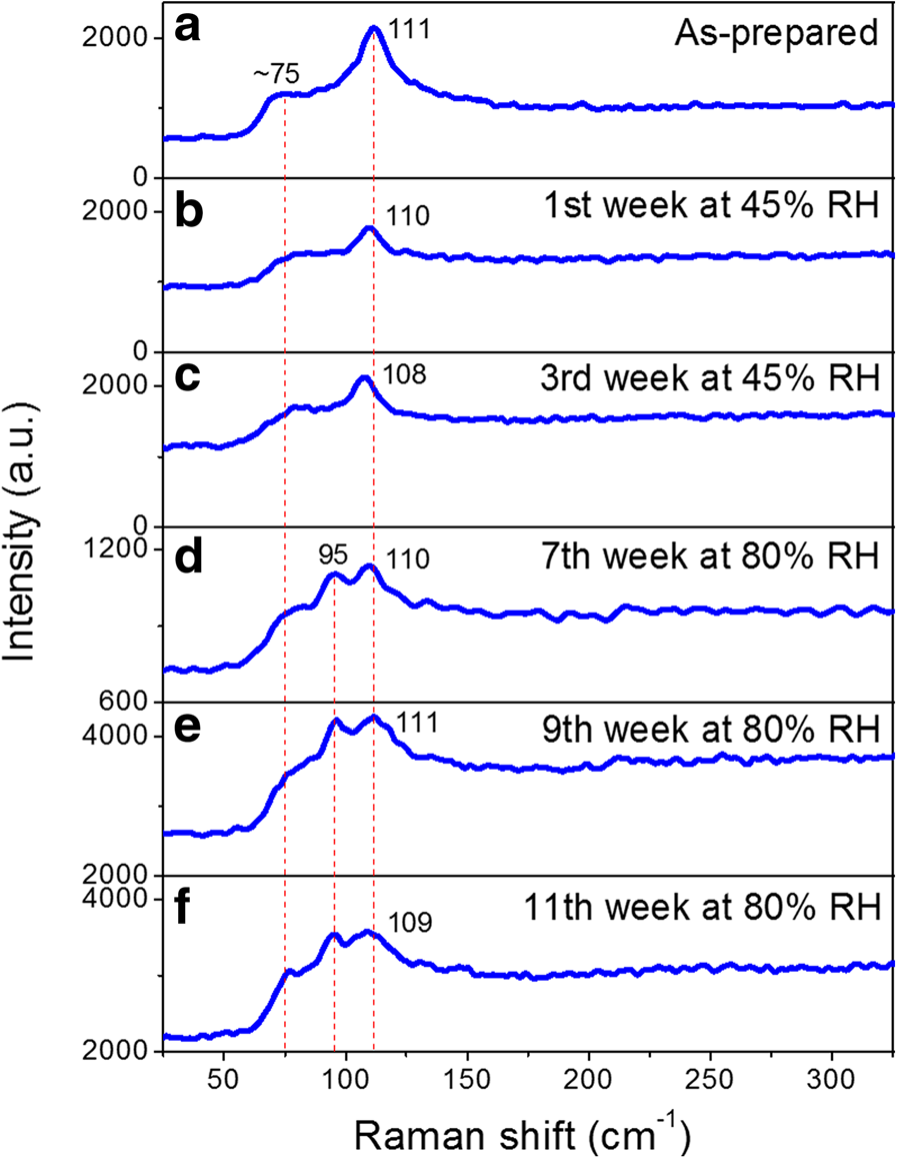
Raman spectra evolution of MAPbI_3_ MWs after humidity exposure. **a** As-prepared sample, after **b** 1 week and **c** 3 weeks at 45% RH and after the **d** 7th, **e** 9th, and **f** 11th weeks at 80% RH. Spectra measured in ambient air with an excitation wavelength of 532 nm, an incident power of 16 μW, an acquisition time of 10 s, and a laser spot diameter of ~ 1 μm on the sample

To study the effect of humidity in the vibrational properties of the MAPbI_3_ MWs, Raman spectroscopy was performed along 11 weeks at different RH levels. The Raman spectra were collected with low laser power of 16 μW at 532 nm to avoid thermal decomposition (see Additional file 1: Figure S3, Raman spectra with higher laser powers). The Raman evolution during the degradation of the perovskite MWs is shown in Fig. 3. Due to the similarity in the vibrational response of different MWs and at different places along the same MW of the as-prepared sample (see Additional file 1: Figure S4), only the Raman profile of one microwire at each stage of degradation is shown. The Raman spectrum of the as-prepared MWs (Fig. 3a) shows a strong peak at 111 cm^−1^ and a shoulder at ~ 75 cm^−1^. A previous Raman study revealed that MAPbI_3_ thin films had two bands at 50 and 110 cm^−1^ [28]. These spectral variations between MWs and thin films may be due to different internal stress levels in the two different morphologies. After the first week at 45% RH (Fig. 3b), the Raman spectrum shows the same two vibrational bands as in the as-prepared sample but with the initial band at 111 cm^−1^ less resolved and shifted to 110 cm^−1^. After prolonging the exposure to 3 weeks at 45% RH (Fig. 3c), the shoulder at ~ 75 cm^−1^ is also observed and the original band at 111 cm^−1^ shifted to 108 cm^−1^. Then, when increasing the humidity to 80% in weeks 7, 9, and 11 (Fig. 3d–f), the Raman spectra revealed a new band at 95 cm^−1^, while the original band at 111 cm^−1^ shifted slightly around its position and the shoulder at ~ 75 cm^−1^ became more resolved in the 11th week.

The Raman profiles of the MWs after exposure to 80% RH (Fig. 3d–f) are comparable to those of PbI_2_ platelets [33], indicating the decomposition of the MAPbI_3_ MWs into the precursor material PbI_2_ solid. However, as we have seen in the “Photoluminescence Evolution of MAPbI_3_ Microwires Under Humidity” section, the photoemission of the MWs after degradation belongs to MAPbI_3_ but not PbI_2_, which indicates that the decomposition of MAPbI_3_ MWs into PbI_2_ is partial. Besides that, the slight position fluctuations observed in the band at 111 cm^−1^ and the appearance of the new band at 95 cm^−1^ upon humidity exposure indicate that the structure of the MWs is locally changed. It is known that H_2_O molecules can incorporate in the crystal lattice solvating the MA^+^ and further dissolve the cations [21], leading to an increment in the density of MA vacancy defects that produces energy levels near the VB [41]. These vacancies can also induce a slight shift of the atoms in the crystal structure that is reflected in the position variation of the Raman mode at 111 cm^−1^. As can be seen in Fig. 3, the band at 111 cm^−1^ shifted to lower frequencies the first 3 weeks, while from the seventh to the ninth week, it shifted to higher frequencies and in the 11th week again towards lower frequencies. In Raman spectra, shifting of peaks to lower vibrational frequencies implies that the corresponding chemical bond length increases, whereas shifting to higher frequencies implies shorter bond length. Previous density functional theory studies on the MAPbI_3_ vibrational properties have related the Raman bands in the range of 70–120 cm^−1^ with Pb–I bond vibration [31, 42]. Thus, the 111 cm^−1^ band shift is due to the stress exerted by H_2_O molecules on the atomic bond corresponding to this vibrational mode of the material and to the atomic shift induced by MA vacancies. However, humidity penetrates at different degrees along the sample because of the heterogeneity of microstructure morphology and defects in the MWs (described in the “Photoluminescence Evolution of MAPbI_3_ Microwires Under Humidity” section of the main text and in Additional file 1: Section 2). This implies that the fluctuation in the position of the 111 cm^−1^ band is probably because the concentration of H_2_O molecules is not the same in the entire sample, which produces different stress levels in the MW bonds and different densities of MA vacancies in the different states of degradation. Therefore, in addition to the increment of the vacancy defects due to the dissolution of MA^+^, humidity could distort the crystal structure of the MWs by the interaction of H_2_O molecules and Pb–I bonds. Besides, the Raman results support the PL redshift of the MWs due to radiative recombination through shallow defects induced by humidity (explained previously in the “Photoluminescence Evolution of MAPbI_3_ Microwires Under Humidity” section). The crystal lattice distortion can be detected with XRD, which is investigated next.

### XRD Evolution of MAPbI_3_ Microwires Under Humidity

**Fig. 4:**
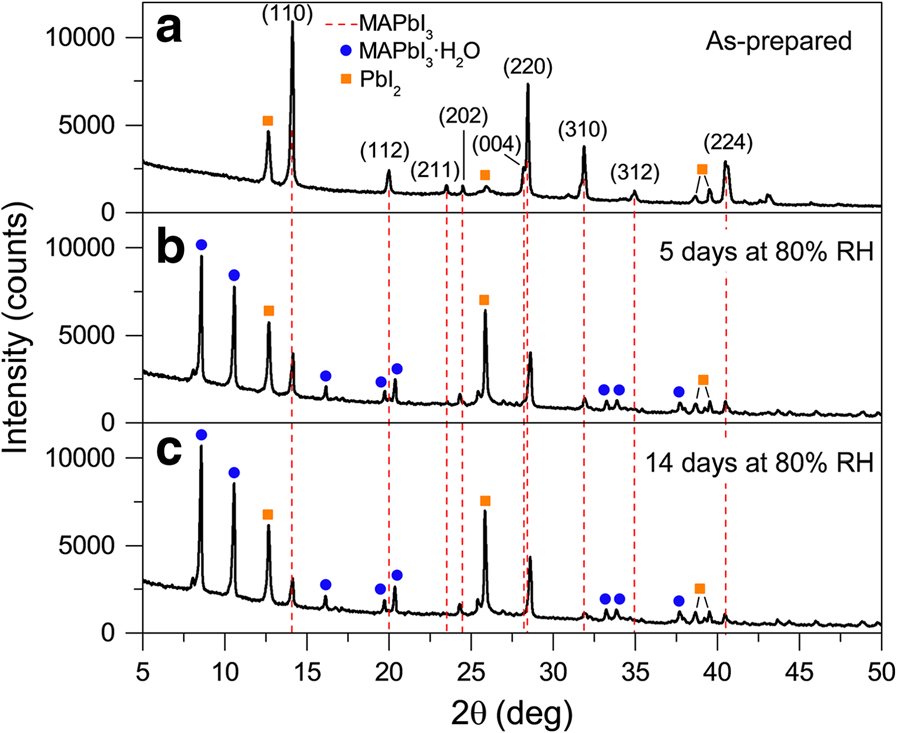
X-ray diffraction pattern evolution of MAPbI_3_ MWs after humidity exposure. **a** As-prepared sample, **b** after 5 days at 80% RH and **c** after 14 days at 80% RH. The red dash lines connecting peaks along the three patterns represent the main perovskite reflections for the tetragonal phase

To elucidate the changes in the crystal structure during the MAPbI_3_ MWs degradation, XRD was performed on a freshly prepared sample and after 5 and 14 days of exposure to 80% RH in the dark. The evolution of the XRD pattern along humidity exposure is shown in Fig. 4. The XRD pattern of the as-prepared MAPbI_3_ MWs is shown in Fig. 4a, and the main diffraction peaks are indexed to the tetragonal phase (as was described in the section “MAPbI_3_ Microwires”). After 5 days of humidity exposure, as shown in Fig. 4b, all the perovskite diffraction peaks (red dash lines) decreased in intensity while the peaks at 2*θ* values of 19.98° and 34.98° vanished completely. Moreover, the reflections belonging to PbI_2_ (orange squares in Fig. 4b) became stronger, confirming the decomposition of MAPbI_3_ into PbI_2_ crystals that was also observed in the Raman spectra. Besides, new reflections (blue circles in Fig. 4b) arose that cannot be assigned to MAPbI_3_, MAI, or PbI_2_, in particular strong peaks at 2*θ* values of 8.54° and 10.54°. Density functional theory calculations and XRD investigations have related these reflections at low angle to the monohydrate phase MAPbI_3_·H_2_O [21, 24, 43]. Moreover, a recent study with multinuclear magnetic resonance of MAPbI_3_ powder at 80% RH determined that the monohydrate phase was the only intermediate hydrate product formed, with no signal of the dihydrate compound even prolonging the exposure to 3 weeks [26]. Thus, we can assign the new peaks to the monohydrate compound MAPbI_3_·H_2_O. As shown in Fig. 4c, prolonging the degradation in humid air to 14 days, the peaks from perovskite decreased slightly in intensity, the peak at 23.50° disappeared, while the PbI_2_ and the hydrate phase reflections barely increased in intensity. In addition, the reflection at 24.50° (Fig. 4a) corresponding to the crystal plane (202) shifted to 24.38° and 24.28° after 5 and 14 days, respectively (Fig. 4b, c). The shift to smaller diffraction angles implies an increment in the lattice plane distance *d*_202_. Meanwhile, the reflections (planes) at 28.19° (004) and 28.45° (220) (Fig. 4a) after 5 days shifted to 28.47° and 28.60° (Fig. 4b), respectively, and without further shift after 14 days of degradation (Fig. 4c). This shift to larger angles implies smaller interplanar spacing *d*_004_ and *d*_220_.

The shift in the XRD peak position observed upon humidity exposure indicates a distortion in the crystal structure of the MWs. It is known that the electronic band configuration of MAPbI_3_ is given by the Pb and I atoms, the upper VB is formed by the *p* orbitals of I, while the lower CB is derived from the *p* orbitals of Pb [44]. In addition, energy gap tunability of hybrid perovskites has been proved with different sizes of organic cations, due to disorder in the crystal structure by tilting the PbI_6_ octahedrons [45, 46]. Furthermore, it has been proposed that in the hydration of MAPbI_3_, the hydrogen bonding interaction of water molecules and the metal halide octahedra is stronger than that from the organic cation [22]. Besides, H_2_O molecules (~ 2.8 Å of diameter) [47] are small enough to penetrate into the MAPbI_3_ MWs crystal structure. Therefore, it is reasonable to suggest that after humidity exposure, water molecules bounded to MA cations inside the MAPbI_3_ MWs lattice could induce distortion to the PbI_6_ frameworks, alter the character of the Pb–I bonds, and thus induce changes in the crystal lattice spacing and vary the optical bandgap. Connecting the XRD results with the PL, we can confirm that the distortion of the MWs crystal structure induced by H_2_O molecules influences the *E*_g_ reduction, the reason why the wavelength of the spontaneous emission redshifted. Hence, in addition to the radiative recombination through shallow trap states induced by humidity, crystal lattice deformation can be another explanation for the MWs PL redshift after humidity exposure. The reduction in the bandgap could lead potentially, for example in solar cells, to higher photon absorption. However, as we have shown that the bandgap reduction after humidity exposure is due to the increment of subgap states (shallow defects) and the distortion of the crystal lattice, the charge carrier dynamics in MAPbI_3_ MW-based optoelectronic devices would be detrimentally affected. The presence of these structural defects may limit the charge transport and collection, for example, thus lessening the device performance efficiency.

## Conclusions

The effect of humidity on the optical and structural properties of MAPbI_3_ MWs was investigated by photoluminescence (PL) spectroscopy, Raman spectroscopy, and X-ray diffraction (XRD). In addition to the common perovskite degradation into PbI_2_ and the monohydrate phase, we have shown that humidity enhanced and redshifted the MWs spontaneous radiative emission. Based on the changes in the Raman bands and XRD reflections, the wavelength redshift of the MWs photoemission was attributed to the structural disorder caused by the incorporation of H_2_O molecules in the crystal lattice and by the radiative recombination through the moisture-induced shallow trap states. The intensity enhancement of PL peaks was attributed to the passivation of nonradiative charge recombination sites (deep trap states) by H_2_O molecules. This study suggests that by controlling the humidity-induced defects and crystal lattice deformation, the optical and structural properties can be preserved, which would improve the material stability and thus the performance efficiency of MAPbI_3_ MW-based optoelectronic devices. At the same time, our results suggest that the photoemission can be tuned by controlling the defect density and the structural deformation of the MWs crystals.

## Additional file

Additional file 1: Additional XRD patterns, calculated lattice parameters, additional PL and Raman spectra, and laser light degradation. (DOCX 3251 kb)

## Abbreviations

CB: Conduction band
DCM: Dichloromethane
DMF: *N*,*N*-Dimethylformamide
MWs: Microwires
PL: Photoluminescence
VB: Valence band
XRD: X-ray diffraction
