# Supplementary material for: Evolution of Photoluminescence, Raman, and Structure of CH3NH3PbI3 Perovskite Microwires Under Humidity Exposure
Source: Nanoscale Res Lett. 2018 Mar 7;13:79. doi: 10.1186/s11671-018-2470-0 (PMC5842165; doi:10.1186/s11671-018-2470-0)
Supplement: Supplementary file 1 — Additional XRD patterns, calculated lattice parameters, additional PL and Raman spectra, and laser light degradation. (DOCX 3251 kb) [file 11671_2018_2470_MOESM1_ESM.docx]

Additional file for

**Evolution of Photoluminescence, Raman, and Structure of CH_3_NH_3_PbI_3_ Perovskite Microwires under Humidity Exposure**

Rubén Segovia^1^, Geyang Qu^1^, Miao Peng^2^, Xiudong Sun^1,3^, Hongyan Shi^1,3*^, Bo Gao^1,3*^

1. Institute of Modern Optics, Key Lab of Micro-optics and Photonic Technology of Heilongjiang Province, Key Laboratory of Micro-Nano Optoelectronic Information System, Ministry of Industry and Information Technology, Department of Physics, Harbin Institute of Technology, Harbin 150001, China.

2. School of Chemistry and Chemical Engineering, Harbin Institute of Technology, Harbin 150001, China.

3. Collaborative Innovation Center of Extreme Optics, Shanxi University, Taiyuan 03006, China.

**1. X-ray diffraction**

Figure S1 shows the X-ray diffraction profile of the as-prepared MAPbI_3_ MWs, the precursor materials used, and a reference. The main Bragg peaks of the tetragonal perovskite structure, space group I4cm (#108),^1^ are identified at 2θ values (crystal planes) of 14.11° (110), 19.98° (112), 23.50° (211), 24.50° (202), 28.19° (004), 28.45° (220), 31.90° (310), 34.98° (312), 40.48° (224), 40.66° (400), and 43.09° (314). The XRD pattern also shows PbI_2_ reflections (orange squares in Figure S1a), with a major peak at 12.67°, indicative of the presence of this precursor material in the final product. The presence of PbI_2_ in the final product has been also observed in MAPbI_3_ polycrystalline films made by different crystallization methods, which was attributed to the incomplete conversion of PbI_2_.^2,3^

Figure S1. X-ray diffraction patterns of a) as-prepared CH_3_NH_3_PbI_3_ MWs on glass slide substrate, b) β-CH_3_NH_3_PbI_3_ (powder, at 293 K) from the crystallography open data base (COD, reference No: 900-5638),^1,4^ and precursor powder materials utilized c) CH_3_NH_3_I and d) PbI_2_. The orange squares represent diffraction peaks assigned to PbI_2_ in the final product. The main crystal planes of the MAPbI_3_ tetragonal phase are identified in a). a), c) and d) Patterns measured in ambient air.

The lattice parameters of the MAPbI_3_ MWs tetragonal structure are extracted from the XRD pattern of the as-prepared sample. Table S1 shows the XRD reflections used for obtaining the unit cell constants (a, b, and c). Bragg’s law (nλ = 2d_hkl_sinθ_hkl_), and the relation between the interplanar spacing d, Miller indices (hkl), and lattice parameters (1/d^2^ = (h^2^+k^2^)/a^2^ + l^2^/c^2^), were used for the calculation of the unit cell dimensions.

Table S1. Calculated lattice constants from peak location and Miller indices of the as-prepared MAPbI_3_ MWs. (λ(Cu-Kα) = 1.5418 Å)

| 2θ (º) | d (Å) | h k l | a = b (Å) | c (Å) |
| --- | --- | --- | --- | --- |
| 14.11 | 6.2721 | 1 1 0 | 8.8701 |  |
| 19.98 | 4.4558 | 1 1 2 | 8.8701 | 12.6612 |
| 23.50 | 3.7855 | 2 1 1 | 8.8701 | 12.6653 |
| 24.50 | 3.6332 | 2 0 2 | 8.8701 | 12.6701 |
| 28.19 | 3.1655 | 0 0 4 |  | 12.6620 |
| 31.90 | 2.8053 | 3 1 0 | 8.8711 |  |
| Average lattice constants = 8.8703 | | | | 12.6646 |

**2. Photoluminescence spectra of three MAPbI_3_ MWs**

Figure S2 shows the PL spectra of three different MWs from the same fresh sample and at three different spots along the same microwire. It can be seen that for different MWs and even at different positions along the same microwire, PL peaks shifted, varied in intensity and in shape. This illustrates the heterogeneity of microstructure morphology and defects in the MWs resulting in the spatially dependent recombination of the photogenerated carriers. The inhomogeneities are related with chemical or structural defects in the material, which are usual in solution-processed hybrid halide perovskites due to the incomplete control during the crystallization.^5,6^

Figure S2. Photoluminescence spectra of three different MAPbI_3_ MWs from the same fresh sample a) Microwire-1, b) Microwire-2, and c) Microwire-3, measured at three different positions along the same microwire (different color in each panel). The figures show the PL peak center (λ_max_) and the full width at half maximum (FWHM) of the Gaussian fitting curves (red lines). Spectra measured in ambient conditions with excitation wavelength of 633 nm, power ~5 µW, laser diameter ~1 µm on the sample, and 10 s of acquisition time.

**3. Raman spectra of three MAPbI_3_ MWs**

Figure S3a shows the Raman spectra of three MAPbI_3_ MWs taken with laser powers of 16, 73, and 148 μW. At 16 μW, the spectrum shows a shoulder at ~75 cm^-1^ and a band at 111 cm^-1^, while the optical image of the microwire does not show any damage after laser exposure (Figure S3b). At 73 μW, the shoulder at ~75 cm^-1^ becomes more significant, and the 111 cm^-1^ band sharper and stronger, implying local damage and a partial decomposition. Figure S3c shows how the color of the microwire changed from grey to yellow after laser exposure. At 148 μW, three new vibrational modes appeared at 97, 163 and 211 cm^-1^. This Raman profile looks alike the profile of MAPbI_3_ film degraded by laser light and with that of PbI_2_,^7-9^ which confirms the decomposition of the MAPbI_3_ MWs by the laser light into the precursor materials (PbI_2_ and MAI). The optical image in Figure S3d shows that the irradiated part of the microwire was almost completely damaged. Moreover, the overheating by the laser can further decompose MAI into CH_3_NH_2_ and HI, which volatilize because of their low boiling points, -6.32 °C for CH_3_NH_2_ and -35.55 °C for HI, leaving only PbI_2_ (872 °C decomposes).^10^

Figure S3. a) Raman spectra of three MAPbI_3_ MWs at different incident laser power. b), c), and d) Corresponding optical images of each microwire after Raman measurement at laser power of 16, 73, and 148 μW, respectively, the red circle indicates the place where the laser beam was pointed at the microwire. Spectra taken in ambient conditions with excitation wavelength of 532 nm, acquisition time 10 s, objective 50x, laser spot diameter of ~1 µm on the sample.

Point out that the Raman spectrum of the degraded microwire with the laser at 148 μW resembles to that of MWs degraded after 80% RH of exposure (Figure 3d-3f in the main text). However, Raman bands at 163 and 211 cm^-1^ (Figure S3a) appeared due to the degradation by laser light did not appear in the spectra of the MWs degraded in humidity (Figure 3d-3f in the main text). This suggests that humidity and laser light induced different degradation mechanisms in the MWs.

The homogeneity of the chemical composition of the MAPbI_3_ MWs was verified performing Raman measurements on three MWs from the same fresh sample, and at three different spots along the same microwire. As shown in Figure S4, the spectra of different MWs and for different position along a single microwire are practically identical, with a shoulder at ~75 cm^-1^ and an intense band at 111 cm^-1^, and with similar band intensities for different MWs. This indicates good uniformity in the material composition along the same microwire and for different MWs from the same as-prepared sample.

Figure S4. Raman spectra of three different MAPbI_3_ MWs from the same fresh sample a) Microwire-1, b) Microwire-2, and c) Microwire-3, measured at three different positions along the same microwire (different color in each panel). All the spectra were collected in ambient conditions with an excitation wavelength of 532 nm, incident power of 16 µW, acquisition time 10 s, objective 50x, and laser spot diameter of ~1 µm on the sample.

**References**

1. Stoumpos, C. C.; Malliakas, C. D.; Kanatzidis, M. G. Semiconducting Tin and Lead Iodide Perovskites with Organic Cations: Phase Transitions, High Mobilities, and Near-Infrared Photoluminescent Properties. *Inorg. Chem.* **2013**, *52*, 9019-9038.

2. Hao, F.; Stoumpos, C. C.; Liu, Z.; Chang, R. P.; Kanatzidis, M. G. Controllable Perovskite Crystallization at a Gas-Solid Interface for Hole Conductor-Free Solar Cells with Steady Power Conversion Efficiency over 10%. *J. Am. Chem. Soc.* **2014**, *136*, 16411-16419.

3. Burschka, J.; Pellet, N.; Moon, S. J.; Humphry-Baker, R.; Gao, P.; Nazeeruddin, M. K.; Gratzel, M. Sequential Deposition as a Route to High-Performance Perovskite-Sensitized Solar Cells. *Nature* **2013**, *499*, 316-319.

4. Grazulis, S.; Daskevic, A.; Merkys, A.; Chateigner, D.; Lutterotti, L.; Quiros, M.; Serebryanaya, N. R.; Moeck, P.; Downs, R. T.; Le Bail, A. Crystallography Open Database (COD): an Open-Access Collection of Crystal Structures and Platform for World-Wide Collaboration. *Nucleic Acids Res.* **2012**, *40*, D420-D427.

5. Rahimnejad, S.; Kovalenko, A.; Fores, S. M.; Aranda, C.; Guerrero, A. Coordination Chemistry Dictates the Structural Defects in Lead Halide Perovskites. *ChemPhysChem* **2016**, *17*, 2795-2798.

6. Peng, W.; Anand, B.; Liu, L.; Sampat, S.; Bearden, B. E.; Malko, A. V.; Chabal, Y. J. Influence of Growth Temperature on Bulk and Surface Defects in Hybrid Lead Halide Perovskite Films. *Nanoscale* **2016**, *8*, 1627-1634.

7. Ledinsky, M.; Loper, P.; Niesen, B.; Holovsky, J.; Moon, S. J.; Yum, J. H.; De Wolf, S.; Fejfar, A.; Ballif, C. Raman Spectroscopy of Organic-Inorganic Halide Perovskites. *J. Phys. Chem. Lett.* **2015**, *6*, 401-406.

8. Zhou, Y.; Garces, H. F.; Padture, N. P. Challenges in the Ambient Raman Spectroscopy Characterization of Methylammonium Lead Triiodide Perovskite Thin Films. *Front. Optoelectron.* **2016**, *9*, 81-86.

9. Preda, N.; Mihut, L.; Baibarac, M.; Baltog, I.; Lefrant, S. A Distinctive Signature in the Raman and Photoluminescence Spectra of Intercalated PbI_2_. *J. Phys.: Condens. Matter* **2006**, *18*, 8899-8912.

10. David R. Lide*, ed., CRC Handbook of Chemistry and Physics, CRC Press, Boca Raton, FL, 2005*.
